# Can Feeding a Millet-Based Diet Improve the Growth of Children?—A Systematic Review and Meta-Analysis

**DOI:** 10.3390/nu14010225

**Published:** 2022-01-05

**Authors:** Seetha Anitha, David Ian Givens, Kowsalya Subramaniam, Shweta Upadhyay, Joanna Kane-Potaka, Yakima D. Vogtschmidt, Rosemary Botha, Takuji W. Tsusaka, Swamikannu Nedumaran, Hemalatha Rajkumar, Ananthan Rajendran, Devraj J. Parasannanavar, Mani Vetriventhan, Raj Kumar Bhandari

**Affiliations:** 1 Smart Food Initiative, International Crops Research Center for the Semi-Arid Tropics (ICRISAT), Hyderabad 502324, India; joanna.kanepotaka@outlook.com; 2 Enabling Systems Transformation (EST), International Crops Research Center for the Semi-Arid Tropics (ICRISAT), Hyderabad 502324, India; s.nedumaran@cgiar.org; 3 Institute for Food, Nutrition and Health, University of Reading, Reading RG6 6EU, UK; d.i.givens@reading.ac.uk (D.I.G.); y.d.vogtschmidt@pgr.reading.ac.uk (Y.D.V.); 4 Food Science and Nutrition, Avinashilingam Institute for Home Science and Higher Education for Women, Coimbatore 641043, India; registrar@avinuty.ac.in; 5 United Nations Children’s Fund (UNICEF), Lilongwe 30375, Malawi; shwetasupadhyay@gmail.com; 6 Development Strategy and Governance Division, International Food Policy Research Institute (IFPRI), Lilongwe P.O. Box 31666, Malawi; rozbotha@gmail.com; 7 Ostrom Center for Advanced Studies on Natural Resources Governance, Asian Institute of Technology, Pathumthani 12120, Thailand; takuji.tsusaka@gmail.com; 8 National Institute of Nutrition (NIN), Hyderabad 500007, India; dirnin_hyd@yahoo.co.in (H.R.); ananthan.nin@gmail.com (A.R.); jpdevraj26@gmail.com (D.J.P.); 9 Gene Bank, International Crops Research Center for the Semi-Arid Tropics (ICRISAT), Hyderabad 502324, India; m.vetriventhan@cgiar.org; 10 National Technical Board of Nutrition, Government of India (GoI), New Delhi 110001, India; rajbhandari53@gmail.com

**Keywords:** height, weight, infant growth, adolescent growth, millets

## Abstract

Undernutrition, such as stunting and underweight, is a major public health concern, which requires multi-sectoral attention. Diet plays a key role in growth and should optimally supply all required nutrients to support the growth. While millets (defined broadly to include sorghum) are traditional foods, and climate smart nutritious crops, which are grown across Africa and Asia, they have not been mainstreamed like rice, wheat, and maize. Diversifying staples with millets can potentially provide more macro and micro nutrients, compared to the mainstream crops. However, there is little known scientific evidence to prove millets’ efficacy on growth. Therefore, a systematic review and meta-analysis was conducted to collate evidence of the benefits of millets in improving the growth of children. Eight eligible randomized feeding trials were included in the meta-analysis. Results from the randomized effect model showed a significant effect (*p* < 0.05) of millet-based diets on mean height (+28.2%) (*n* = 8), weight (*n* = 9) (+26%), mid upper arm circumference (*n* = 5) (+39%) and chest circumference (*n* = 5) (+37%) in comparison to regular rice-based diets over for the period of 3 months to 4.5 years, which was based on largely substituting rice with millets. When an enhanced and diverse diet was served, replacing rice with millet had only minimal growth improvement on chest circumference (*p* < 0.05). The quality assessment using GRADE shows that the evidence used for this systematic review and meta-analysis had moderate quality, based on eight scoring criteria. These results demonstrate the value of adding millet as the staple for undernourished communities. Further understanding of the efficacy of millets on growth in a wider range of diets is important to develop appropriate dietary programs and improve the nutritional status of various age groups across Africa and Asia.

## 1. Introduction

Undernutrition, especially stunting, underweight, and wasting, is a major global crisis. It is estimated that there are currently 149 million children under five years who are affected by stunting, and 45 million children under five years who are affected by wasting [1]. Undernutrition puts children in greater risk of susceptibility to infections, increases the frequency and severity of such infections, and delays recovery. Approximately, 45% of global deaths among children under five are linked to undernutrition [2], which is particularly common in low- and middle-income countries. It is noteworthy, over a half of the stunted children under five years live in Asia, and more than one-third in Africa, while more than two-thirds of the wasted children live in Asia and more than one-quarter in Africa (UNICEF/WHO/World bank, 2019). 

After birth, the first 1000 days of life are a critical stage and a window of opportunities for healthy growth, including physical, social, emotional, and cognitive development [3]. Similarly, the adolescent stage is another important growth period in the human life cycle. Although several other factors affect growth (e.g., infection), diet is a major factor which cannot be neglected. Nutrient deficient diets hinder children’s short and long-term physical, mental, and emotional development, affecting not only individual development but also economic and social development of the country. India’s latest National Family Health Survey indicates stagnation in most indicators related to the nutritional status of children including underweight, wasting, stunting and iron deficiency anaemia [4].

Child malnutrition is considered as a sensitive indicator of the overall levels of food insecurity and hunger. The Sustainable Development Goal 2 (SDG 2) aims to eliminate hunger and all forms of malnutrition by 2030. Diversified diet, especially staples, are needed to address the global challenge of hidden hunger. More than 70% of the energy intake comes from Big 3; rice-, wheat-, and maize-based foods in developing countries of Africa and Asia [5,6]. The traditional crops, such as millets, are rich in nutrients including protein, iron, and zinc. Finger millets are particularly rich in calcium along with other nutrients that are generally lacking in other staples, such as milled rice, refined wheat, and maize [7].

Many nutrients are essential during the growth and development stages, especially for linear skeletal growth. These include adequate supply of energy, amino acids, and bone forming minerals such as calcium, phosphorus, magnesium, and zinc and other ions and vitamins, such as vitamin C, D, and K required for collagen formation and bone metabolism and/or phosphate homeostasis [8].

Given the high nutrient content of millets, understanding their role in child growth would be instrumental in achieving sustainable nutritional security. Millets secure the sixth position in global production of cereal grains and remain a staple food in many rural marginalized regions of the world [9]. With rich sources of many vital nutrients, they have promising potential to combat nutrient deficiencies in third world countries. In addition, millets are recognized as smart foods [10] as they are “good for you” (nutritious and healthy), “good for the planet” (e.g., can survive with less water and other inputs and have a low carbon footprint), and “good for the farmer” (e.g., survive in high temperatures and resilient). However, their beneficial effects are often neglected, especially their impact on growth, as reported in scientific studies decades ago [11].

India is one country proactively striving to bring millets back as common part of the diet. The Government of India launched the national millet mission in 2018 and celebrated that year as the ‘National Year of Millets’. The National Institution for Transforming India (NITI) Aayog announced a pilot to include millets in the Integrated Child Development Services (ICDS) and Mid-Day Meal (MDM) schemes across the country. The year 2023 has been approved by the United Nations as the International Year of Millets, which is expected to attract major attention to millets. Previous systematic reviews and meta-analyses conducted on millets’ health and nutritional benefits showed that regular consumption of millets could help manage the risk of developing type 2 diabetes [12], reduce hyperlipidaemia, hypertension, and body mass index (BMI), thereby helping manage cardiovascular disease risk [13], improve haemoglobin level, and reduce anaemia [14], as well as helping calcium retention [15]. However, scientific evidence of millets’ role in addressing global crises, such as impaired growth, has had fewer studies undertaken in order to prioritize these crops in nutritional interventions. This is the first systematic review and meta-analysis aimed to collate all the evidence from controlled feeding trials that has been conducted to assess the impacts of consumption of millet-based diets on the growth of children.

Review question: Does consumption of millet-based diets help improve child growth (infant, school going, and adolescent) compared to non-millet diets?

## 2. Materials and Methods

The study adopted a 27-item PRISMA checklist [16,17] for every step in the data collection, extraction, and analysis. The systematic literature review process was started in October 2017 and concluded in February 2021. As there were limited studies on this subject, all the studies published until 2019 on millets and their impacts on growth were included regardless of which year the study had been conducted. The protocol for this systematic review and meta-analysis was registered retrospectively in the online registration platform (www.researchregistry.com), with the unique identification number (UIN), Reviewregistry1180, which can be accessed by browsing the registry of systematic review/meta-analyses with UIN.

### 2.1. Search Strategy

Studies published in English were considered. Search engines Google, Scopus, Web of Science, PubMed, and CAB abstract were used to identify relevant studies. The search key words are given in Table 1. Articles were screened for relevance of the scope, completeness of information, and quality of the research, based on the inclusion and exclusion criteria. Further searching was undertaken within library hard copies and scientific community contacts. The identified papers were used only if they addressed the research question, “Does consumption of millet-based diets help improve child growth (infant, school going, and adolescent) compared to non-millet diets?”. If the abstract was found suitable, efforts were made to download the open access articles or collect the full papers from the library. After collecting the full papers, if any relevant data were missing, the authors of relevant published articles were contacted to obtain additional data. A hand search was used on the reference list of every eligible article to find further related research articles.

### 2.2. Eligibility Criteria

Inclusion criteria: 1. Randomized controlled trials conducted to test the efficacy of millets on growth; 2. Studies that assessed at least one of the following growth outcomes: height, weight, mid upper arm circumference (MUAC), chest circumference, body mass index (BMI), height for age *Z*-score (HAZ), and weight for age *Z*-score (WAZ); 3. Studies conducted in populations at different stages of growth including infants (<23 months), pre-school children (<59 months) school going children (5 to 10 years), and adolescents (11 to 19 years) by feeding millet-based diets and with their anthropometry measured; and 4. Only peer reviewed journal articles were included.

Exclusion criteria: 1. Review articles were excluded for further consideration; 2. Animal studies were excluded; and 3. If the data were incomplete, the authors were contacted for missing and/or additional information. The minimum data required were changes in mean and standard deviation (SD) of height, weight, MUAC, and chest circumferences. In case, if the data were still incomplete (for example the change in SD was not available), even after contacting the authors then the study was excluded.

### 2.3. Study Selection and Data Extraction

Two independent reviewers (S.A and S.U) extracted data. Each study was labelled with author details and year. Trial participant characteristics such as age group and gender were recorded, along with the country in which the study was conducted, study design, sample size, duration, type and form of millet used, and outcomes assessed such as height (cm), weight (kg), MUAC (cm), and chest circumference (cm) for millet consuming group (intervention) and the regular diet or enhanced rice-based diet consuming group (control). The data were then entered into an Excel sheet as per the guidelines provided by Harrer et al. (2019) [18]. The effect of millet-based diets on growth parameters were captured as the difference in differences (DID), which is the differences in changes in mean and SD of measurement from the pre intervention to post intervention between the intervention group (millet consuming group) and the control group (non-millet consuming group or enhanced rice-based diet group). If the paper already provided the changes in mean and SD from the baseline, then these values were extracted. If the changes in mean and SD were not provided, then these values were calculated using the reported data. The changes in mean were calculated using the following formula: Mean_change_ = Mean_postintervention_ − Mean_pre-intervention_. The changes in SD were calculated using the method and formula provided in the online Cochrane handbook with the topic on imputing standard deviations for changes from baseline [19]. In brief SD changes were calculated from the t value if it was provided in the manuscript using the formula

$$\mathrm{SE}=\frac{\mathrm{MD}}{t}$$

where SE is standard error, and MD is difference in means. Once SE was obtained then it was used in the formula below to obtain SD:

$$\mathrm{SD}=\frac{\mathrm{SE}}{\sqrt{\frac{1}{N_{E}}+\frac{1}{N_{C}}}}\;$$

where N_E_ and N_C_ are sample sizes in the intervention and control groups respectively. If the t statistic was not provided, then SD was calculated using correlation formula as given below. Correlation for both groups was calculated and the mean correlation value was then obtained (E stands for experiment, equivalent to intervention in our study, which is replaced by C for the control).

$${\mathrm{Corr}}_{E\;}=\frac{{\mathrm{SD}}_{E,\mathrm{baseline}\;}^{2}+{\;\mathrm{SD}}_{E,\mathrm{final}\;}^{2}-{\;\mathrm{SD}}_{E,\mathrm{change}}^{2}}{2\times {\;\mathrm{SD}}_{E,\mathrm{baseline}\;}\times {\;\mathrm{SD}}_{E,\mathrm{final}}}$$

Correlation value was then used in the below formula for both groups. Again, E is replaced by C when calculating the difference for the control group).

(1) $${\mathrm{SD}}_{E,\mathrm{change}\;}=\sqrt{{\mathrm{SD}}_{E,\mathrm{baseline}}^{2}+{\;\mathrm{SD}}_{E,\mathrm{final}}^{2}-(2\times \mathrm{Corr}\;\times {\;\mathrm{SD}}_{E,\mathrm{baseline}}\times {\;\mathrm{SD}}_{E,\mathrm{final}\;}}\;$$

### 2.4. Risk of Bias Assessment

Funnel plot was used to visually assess the presence of publication bias, and other bias such as selection, detection, attrition, and reporting bias following the guidelines provided in the Cochrance handbook for systematic reviews of interventions [19].

### 2.5. GRADE to Assess the Quality of the Evidence

The quality of the evidence was assessed using eight assessment criteria as described by Cochrane author resources using the GRADE approach. The GRADE was not included during the protocol writing stage. During the drafting and review stage, the GRADE was included based on a reviewer’s comment. As per the guidance, two authors of this systematic review independently conducted the GRADE assessment. At any point, when there was disagreement on a particular item, another author of this paper was involved in the assessment for final decision. All the outcomes underwent an overall assessment rather than an independent assessment as they all originated from the same studies. The quality was assessed and rated based on the following criteria; 1. risk of bias, 2. inconsistency, 3. indirectness, 4. imprecision, 5. publication bias, 6. large magnitude of effects, 7. dose response, and 8. effects of all plausible confounding factors. Ratings were given to downgrade the first five criteria and/or upgrade the sixth to eighth criteria based on the assessment for each criterion.

### 2.6. Summary Measures and Result Synthesis

The changes in mean and SD of height, weight, MUAC, and chest circumference from the baseline to the end-line for the intervention group and the control group were compared in the meta-analysis to measure Standard Mean Difference (SMD) and heterogeneity (i^2^). The significance of the results was determined using the fixed effect model for single source information, specifically when all the studies were conducted by one research team from one geographical location and SMD was not largely different between the fixed and random effect models, and when the heterogeneity was less than 50%. On the other hand, the random effect model was used mainly to compare finger millet-based diets vs. rice-based diets (regular and enhanced), and when heterogeneity was more than 50% to interpret the results. Results from both models were captured in each forest plot. Meta-analysis was conducted using the software R Studio version 4.1.1 (2021) to obtain forest plot along with heterogeneity (i^2^) and overall test effects in both models and funnel plots to determine the publication bias [20,21,22,23,24].

## 3. Results

**Figure 1:**
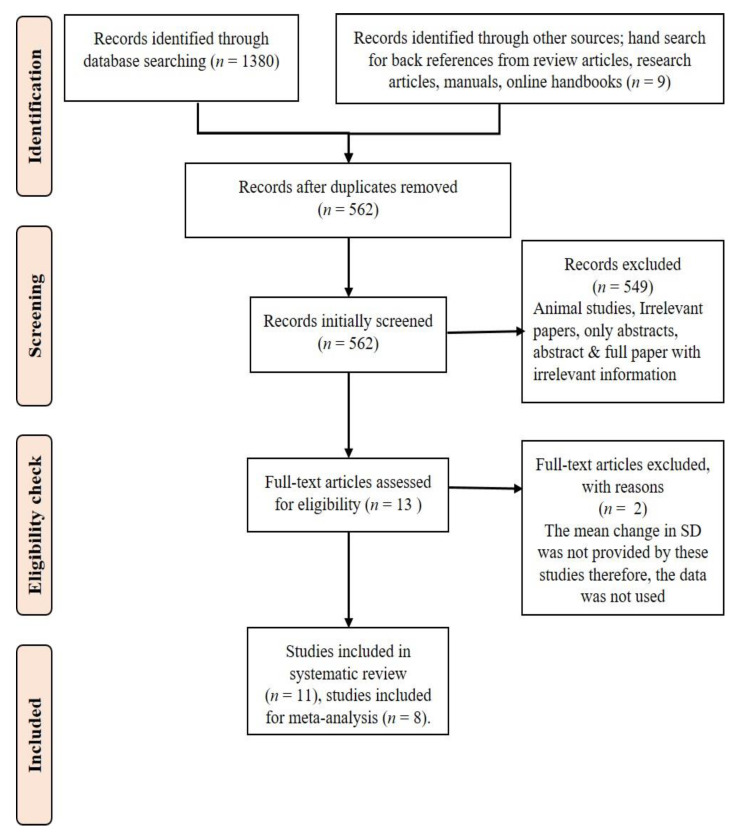
PRISMA flow diagram for the systematic review.

Figure 1 is a PRISMA flow diagram depicting the different stages of the systematic review with the number of records identified, included, and excluded.

Study characteristics are given in detail in Table 2 which includes the author information with year of study, country, study participants, sample size, study period, parameters, study design, and remarks.

### 3.1. Mean Change in Height (Millet Diet vs. Regular Rice Based Diet)

**Figure 2:**
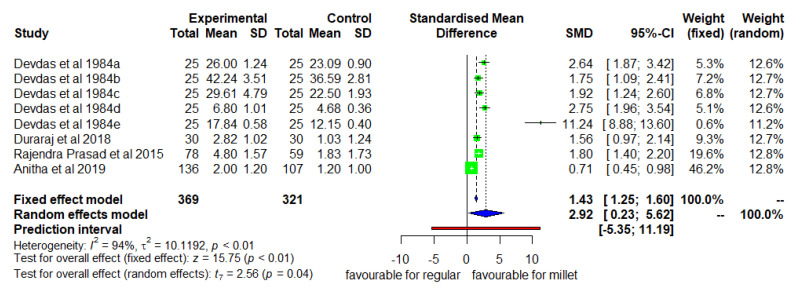
Effect of consuming a millet-based diet on mean height change compared to a regular rice-based diet.

Figure 2 shows the mean change in height from the baseline to the endline as a result of consumption of millet vs. regular rice-based diets. The DID was positive and significant (*p* < 0.05) with high heterogeneity (I^2^ = 94%) among studies and a pooled SMD of 2.92 (95% CI 0.23–5.62). The mean height increase was 28.2% higher in the intervention group than in the control group.

### 3.2. Mean Changes in Weight (Millet Diet vs. Regular Rice-Based Diet)

**Figure 3:**
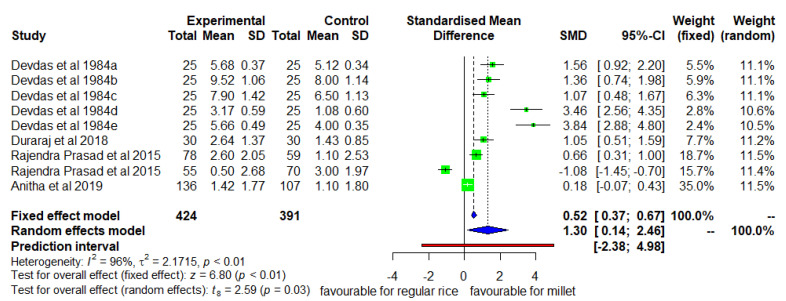
Effect of consuming a millet-based diet on mean weight change compared to a regular rice-based diet.

Figure 3 shows the mean changes in weight from the baseline to the endline for the intervention and control group. The DID was positive and significant (*p* < 0.05) with high heterogeneity (I^2^ = 96) among the studies and a pooled SMD of 1.30 (95% CI 0.14; 2.46) (Figure 3). The mean weight increase was 25% higher in the intervention group than in the control group.

### 3.3. Mean Changes in MUAC (Millet Diet vs. Regular Rice Based Diet)

**Figure 4:**
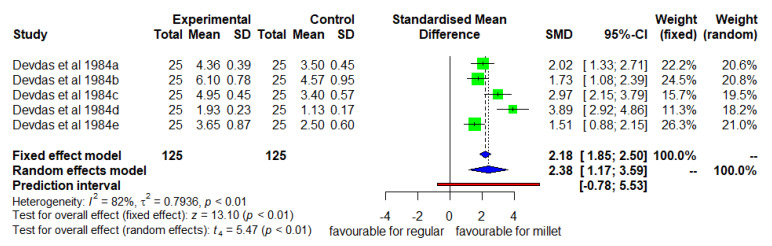
Effect of a consuming finger millet-based diet on mean change in MUAC compared to a regular rice-based diet.

Likewise, Figure 4 shows the mean changes in MUAC for the two groups. The DID was positive and significant (*p* < 0.05) with high heterogeneity (I^2^ = 82%) among studies and SMD of 2.38 (95% CI of 1.17; 3.5). The mean increase in MUAC was 39% higher in the intervention group than in the control group.

### 3.4. Mean Changes in CHEST Circumference (Millet Diet vs. Regular Rice-Based Diet)

**Figure 5:**
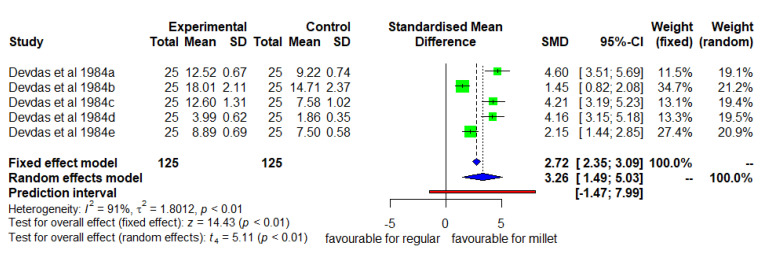
Effect of consuming a finger millet-based diet on mean change in chest circumference compared to a regular rice-based diet.

Figure 5 shows the mean change in chest circumference. The DID was positive and significant (*p* < 0.05) with high heterogeneity (I^2^ = 91%) and SMD of 3.26 (95% CI of 1.49; 5.03). The mean increase in chest circumference was 37% higher in the intervention group than in the control group.

### 3.5. Mean Changes in Height (Enhanced Diverse Millet Diet vs. Enhanced Diverse Rice Diet)

**Figure 6:**
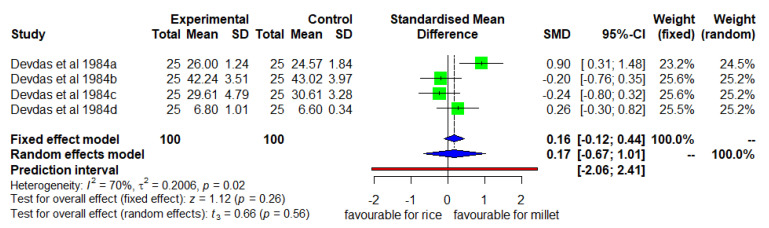
Effect of consuming an enhanced diverse millet-based diet on mean height change compared to an enhanced diverse rice-based diet.

Figure 6 shows the means changes in height for the intervention group consuming an enhanced diverse millet diet and the control group consuming enhanced diverse rice diet. The DID was found to be statistically insignificant (*p* = 0.26) with moderate heterogeneity (I^2^ = 70%) among studies and SMD of 0.16 (95% CL of −0.12; 0.44).

### 3.6. Mean Changes in Weight (Enhanced Diverse Millet Diet vs. Enhanced Diverse Rice Diet)

**Figure 7:**
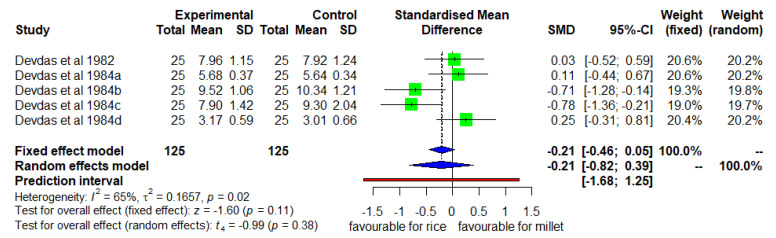
Effect of consuming an enhanced diverse millet-based diet on mean weight change compared to an enhanced diverse rice-based diet.

Figure 7 shows the mean changes in weight for the intervention and control group. The DID was insignificant (*p* = 0.11) with moderate heterogeneity (I^2^ = 65%) among studies and SMD of −0.21 (95% CL of −0.46; 0.05).

### 3.7. Mean Changes in MUAC (Enhanced Diverse Millet Diet vs. Enhanced Diverse Rice Diet)

**Figure 8:**
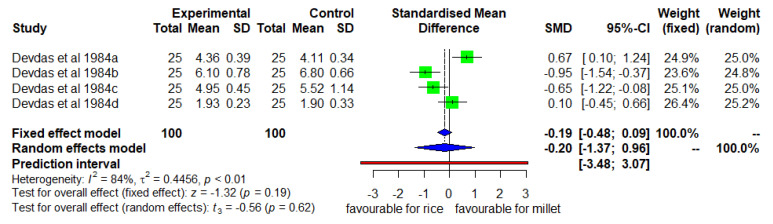
Effect of consuming an enhanced diverse millet-based diet on mean MUAC change compared to an enhanced diverse rice-based diet.

Likewise, Figure 8 shows the mean changes in MUAC for the two groups. The DID was insignificant (*p* = 0.19) with high heterogeneity (I^2^ = 84%) among studies and SMD of −19 (95% CL of −0.48; 0.09).

### 3.8. Mean Changes in Chest Circumference (Enhanced Diverse Millet Diet vs. Enhanced Diverse Rice Diet)

**Figure 9:**
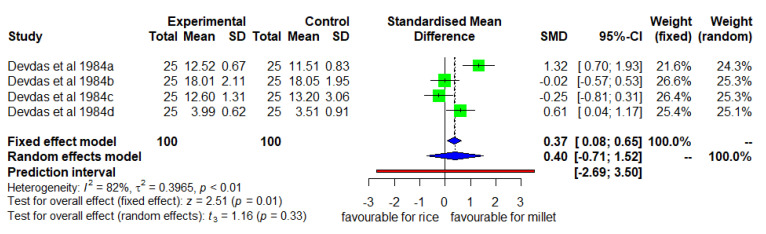
Effect of consuming an enhanced diverse millet-based diet on mean chest circumference change compared to an enhanced diverse rice-based diet.

Figure 9 shows the mean changes in chest circumference. In the fixed effect model, the DID was positive and significant (*p* < 0.01) high heterogeneity (I^2^ = 82%) among studies and SMD of 0.37 (95% CL of 0.08; 0.67). However, the mean increase in chest circumference was only slightly higher (2%) in the intervention group consuming a finger millet than in the control group consuming a rice-based diet.

### 3.9. Publication Bias and Risk of Bias

The publication bias was determined through the funnel plot (Supplementary Figures S1–S8) using the trim and fit model. Some funnel plots were asymmetrical, which suggests some publication bias. Risk of bias assessment (Supplementary Figure S9) indicates moderate risk of bias coming from evidence used in the meta-analysis. This is further elaborated in the discussion section.

### 3.10. Effect of Interventions

All the outcomes were looked at for GRADE assessment. The overall effect of the interventions shows that the quality of the evidence is moderate as there is moderate confidence in the effect estimate. This implies that the true effect is likely to be close to the estimate of the effects, though there is a possibility of being substantially different.

## 4. Discussion

### 4.1. Publication Bias, Risk of Bias, and Quality of Evidence

Most studies included in this meta-analysis randomized the children allocation to the treatment and control groups. However, they generally did not mention how the randomization was undertaken or whether the experiments were blinded or not. Nevertheless, the studies reported that the participants in both the intervention and control groups were similar at the baseline in terms of socio-economic status and dietary patterns. It is noteworthy that it is almost impossible to blind participants for the millet-based meals that are provided during these studies, as millets are unique in their appearance, colour, and texture, which can be identified by anyone in the study areas where millets are traditional crops. Similarly, the anthropometry measurements cannot be blinded like clinical measurements. Most clinical measurements collect biological samples, such as blood, whereas anthropometry measurements involve measuring height, weight, MUAC, and chest circumferences, which are visible to the participants. As these is not blinding, there is no risk of bias arising from blinding per se. Only allocation concealment can be considered as a small risk coming from the studies for GRADE assessment.

The heterogeneity (I^2^) value for various outcomes varied from 65% to 96%. The high heterogeneity could be due to clinical heterogeneity associated with differences among participants in different stages of growth (infants, children, and adolescents). There is also a possibility of methodological heterogeneity as the duration of each of the studies varied from 3 months to 4.5 years. Except for one study conducted for 3 months and another for 6 months, all other studies were conducted for longer period from 8 months to 4.5 years. Therefore, the high statistical heterogeneity in the outcomes could be associated with the differences in growth stages and study duration. Subgroup analysis could not be performed due to the small number of studies in each group.

All the studies included in the meta-analysis directly contributed to the review question of this systematic review. Therefore, there was no indirectness identified during GRADE assessment. The observed publication bias could have come from the small number of studies and their heterogeneity.

The growth effects of millet-based meals in comparison with normal non-enhanced rice-based meals were positive and significant (Figure 2, Figure 3, Figure 4 and Figure 5), indicating that the magnitude of the effects shows strong association, and it was hence upgraded in GRADE assessment. Due to some imprecision and publication biases observed, the quality of the evidence used is rated as moderate. While the evidence remains highly useful, it is recommended to conduct more to increase the overall number of studies and have more recent results, given that except for two studies, all were undertaken decades ago.

Most of the studies included in the current systematic review and meta-analysis investigated finger millet apart from one study with sorghum and two with a mixture of millets (finger, pearl, foxtail, little, and kodo millets). Overall, two types of studies were conducted—substituting rice for millet in regular diets and substituting rice for millets in enhanced diverse diets. The studies that formulated the enhanced diverse diets (millet or rice) took into account the nutrient gaps identified in the dietary patterns of the people.

Nutrient requirements generally vary by age. During adolescence, typically the weight doubles and height grew 20% in healthy individuals [34]. During this time all the essential nutrients are required, especially protein, calcium, iron, and vitamins, to support bone accretion and to enhance the growth. Protein, especially branched chain amino acid leucine and sulphur containing amino acids, is a key nutrient required to support the development of muscle mass, and therefore, are especially important during adolescence [34]. Calcium always remains as a priority nutrient for bone growth especially during the tremendous growth periods of infancy (first twelve months) and adolescents (13 to 18 years).

Finger millets have a threefold higher concentration of calcium than milk, and calcium retention is higher for finger millet than for any other staple [15]. Moreover, millets contain more methionine, sulphur amino acids, compared to milled rice and refined wheat [7].

In the regular diets, there was a significant positive effect of replacing rice with millet on height, weight, MUAC, and chest circumference in various age groups (infant, pre-school, school going, and adolescents). These positive effects of millets are attributed to the naturally high content of growth promoting nutrients (especially sulphur amino acids, total protein, and calcium in case of finger millets), given that the rest of the diet was similar between two groups.

Table 3 has information [27,28] on food provided in the interventions with preschool and school-going children, showing the composition of the diets based on diversified and enhanced rice and diversified and enhanced finger millet, compared to regular rice-based diets. It is noted that the enhanced diets based on finger millet had higher quantities of each food group: with 45% more finger millet, 270% more pulse, 59% more dairy products, 140% more green leafy vegetables, and 64% more fruits. On the other hand, the enhanced rice-based diets had higher quantities of each food group: 35% more rice, 327% more pulse, 59% more dairy products, 194% more green leafy vegetables, and 70% more fruits, compared to the regular rice-based diets. This likely explains the reason for the significant growth observed in the groups fed with the enhanced finger millet and enhanced rice-based diets, compared to the controlled regular rice-based diet group. On the other hand, there was no significant difference between the enhanced finger millet and enhanced rice diets in terms of consumption quantity per day, i.e., they were similar diets, with the main exception being a substitution of rice with millet. There was no significant difference between enhanced finger millet and enhanced rice-based diets in any of their growth parameters except chest circumference, which received a slight increase in growth (2%) in the enhanced finger millet-consuming group.

This shows, that when the diet is enhanced, diverse, and nutritious, there is only minimal extra growth achieved from consumption of millet-based diets compared with rice-based diet. This is a reasonable result given that the control diet contains a diverse range and adequate quantities of nutritious foods. However, when the base diet was not enhanced and had lower nutritional value, substituting rice with millet had a large and significant impact on additional growth [6,32,33]. This provides evidence that including millets as the staple in malnourished communities can significantly improve the health and hence growth of the people.

### 4.2. Limitations and Future Research Recommendations

There are nine main limitations in this study as follows. 1. The studies that were eligible for meta-analysis included finger millet, sorghum, and multiple millets (finger millet, pearl millet, and little millet). The other types of millets should be studied to determine which millets have the most positive impact on growth, and hence the most important to be included in nutrition sensitive agriculture interventions and programs. 2. All the studies included in the meta-analysis were from India, which indicates a geographical limitation and a need to conduct such studies in other countries, especially across Africa where millets are traditional crops and there are serious malnutrition issues. 3. Further studies should be undertaken including all the growth measurements. Two studies measured the BMI, and other studies measured only height and weight without estimating the BMI, HAZ, and/or WAZ, which could have been included to study the stunting and underweight status. 4. The studies had similar diets and mainly only substituted the staple. However, the diets were not exactly the same and thus future studies should design the intervention by keeping all food groups exactly the same between the intervention and control groups, changing only the selected staple (e.g., replace rice with millet). 5. All major growth promoters should be measured in future studies. Methionine is an essential sulphur containing amino acid and a major growth promoter. However, none of the studies measured the level of methionine provided in the diets in both groups. Apart from methionine, total protein, calcium, and zinc are essential as growth promoters, which were also not measure. 6. Growth impacts of the different types of cooking/processing of the millets should be analysed to inform more detailed recommendations for dietary design. 7. No studies identified the varieties of millets used or the nutritional content of the grain or the total diet. Studies incorporating these factors would help identify how important the varieties are on growth outcomes. 8. Only one study was designed to analyse the impacts of millet-based diets on children’s growth during adolescence, the third critical stage of rapid growth and bone mineralization in human life, which calls for further studies. 9. Except two studies, all other studies were conducted in the 1980s by one research team, suggesting that new studies should be undertaken to corroborate the results.

### 4.3. Policy Recommendations

The developmental, economic, social, and medical impacts of the global burden of malnutrition are serious and lasting for individuals, their families, communities, and countries [35]. Hence, nutritional interventions should be one of the priority areas for policy-makers, especially where the burden is high.

These studies provide evidence that millet-based diets can be effective in improving height and weight where regular rice-based diets are currently consumed. At the same time, these studies were not aimed at making changes in dietary patterns. Policy recommendations include: 1. Nutrition intervention programs developed to diversify staples using millets. 2. School feeding programs, and mother and child programs, incorporate millet-based meals designed for different age groups, using with culturally sensitive and tasty recipes. 3. Complement these interventions with awareness and marketing campaigns to change the image of millets and create interest. Implementation of these recommendations should be studied in regards to the impact and lessons learnt from the approaches.

## 5. Conclusions

The findings of this review paper provide scientific evidence supporting diversification of standard diets for children using millets as a solution to malnutrition. Furthermore, millets are a basket of a wide range of nutrients and have been scientifically shown to contribute to serving many of the significant nutrition and health needs globally [12,13,14,15], not only by tackling child undernutrition, but also by managing type 2 diabetes [12], lowering total cholesterol levels, obesity [13], and iron deficiency anaemia [14]. To bring this solution to reality, awareness about the nutritional value is needed to drive demand and investments in millets along the value chain, from fork to farm.

## Figures and Tables

**Table 1:** Key words used for the literature search.

Number	Keywords
1	Boolean logic such as “AND”, “OR”, “NOT” were used
2	Millets efficacy on child growth
3	Effect of millet supplementation on growth of the “children” OR “adolescents” OR ”pregnant women”
4	Effect of “millet” OR “finger millet” OR “pearl millet” OR “sorghum” OR “little millet” OR “barnyard millet” OR “Job’s tears” supplementation on growth of the children AND adolescent AND infant AND pregnant women

**Table 2:** Characteristics of the studies evaluated for final inclusion in the systematic review and meta-analysis.

Author	Country	Type of Millet	Form Consumed	Study Participants	Duration	Sample Size	Parameters Studied	Study Design and Remarks
Devdas et al. (1982) [25]	India	Finger millet	Meal	Pregnant women	9 months	25 interventions (finger millet-based diet), 25 control	Weight, haemoglobin	Controlled feeding trial. This study was not included for meta-analysis as it focused on pregnant women.
Devdas et al. (1983) [11]	India	Finger millet	Meal	Nursing mother	0–18 months	25 interventions (finger millet-based diet), 25 control	Weight, haemoglobin	Controlled feeding trial. This study was not included in meta-analysis as it focused on lactating mother.
Devdas et al. (1984a) [26]	India	Finger millet	Finger millet malted, finger millet porridge, cooked with pulses, Idly, adai	Infants	0–18 months	25 interventions (finger millet-based diet), 25 control	Weight, height, chest circumference, mid upper arm circumference	Controlled feeding trial by feeding a millet-based diet to one group, enhanced rice-based diet to another group and the third group is a control which consumed a regular rice-based diet.
Devdas et al. (1984b) [27]	India	Finger millet	Meal	Preschool children	0–4 years	25 interventions (finger millet-based diet), 25 control	Weight, height, chest circumference, mid upper arm circumference	Controlled feeding trial by feeding millet-based diet to one group, enhanced rice-based diet for another group and third group is a control which was consuming a regular rice-based diet.
Devdas et al. (1984c) [28]	India	Finger millet	Meal	School children	2.5 to 4.5 years	25 interventions (finger millet-based diet), 25 control	Height, weight, chest circumference, haemoglobin, serum protein level	As described above [27]
Devdas et al. (1984d) [29]	India	Finger millet	Meal	School children	6 to 7.5 years	25 interventions (finger millet-based diet), 25 control	Height, weight, chest circumference, haemoglobin	As described above [27]
Devdas et al. (1984e) [30]	India	Finger millet	Meal	Preschool children	0 to 3 years	25 interventions (finger millet-based diet), 25 control	Weight, height, chest circumference, arm circumference, haemoglobin level	As described above [27]
Devdas et al. (1984f) [31]	India	Finger millet	Meal	Preschool children	3.5 to 6.5 years	25 interventions (finger millet-based diet), 25 control	Height, weight, chest circumference, haemoglobin	Controlled feeding trial. This was not included in meta-analysis as baseline values were missing to calculate mean difference.
Durairaj et al. (2018) [32]	India	Kodo, little, foxtail	health drink	Primary school children	6 months	30 interventions, 30 control	Height and weight	Controlled feeding trial. SD changes were calculated using t statistics provided in the paper as per Cochrance handbook.
Rajendra Prasad et al. (2015) [33]	India	Sorghum	Sorghum roti (flat bread), cooked with water same as rice, Khichidi/upma	School going children	8 months	78 interventions	Height, weight, haemoglobin, BMI	Controlled feeding trial tested a sorghum-based diet in one group (intervention) and an enhanced rice-based diet in another group (control). This study was included for weight parameters. For the height parameter, the boy’s group value was excluded as it was presented in two papers in two different ways by the same authors, which influenced the entire study.
Anitha et al. (2019) [6]	India	Finger millet, pearl millet, little millet	Kchichidi, ragi idly, bisibelle bath, little millet cooked with water as a rice	Adolescents	3 months	136 interventions, 107 control	Height for age and BMI for age	Controlled feeding trial which fed a millet-based diet to an intervention group and an enhanced rice-based diet to a control group. Height and weight values were obtained from the authors.

**Table 3:** Quantity of food items fed per day to groups consuming enhanced finger millet, enhanced rice and regular rice diets.

Food Items (g/day)	Enhanced Finger Millet Based Diet	Enhanced Rice Based Diet	Control Diet (Regular Rice Based Diet)
	Mean ± SD	Mean ± SD	Mean ± SD
Millet/rice (g)	245 ± 20.4	228.3 ± 26.3	168.7 ± 26.2
Pulses (g)	100 ± 8.6	115.3 ± 5.0	27.0 ± 11.2
Milk & milk products (g)	54.2 ± 6.7	54.0 ± 9.6	34.0 ± 6.4
Roots and tubers (g)	96.7 ± 25.7	113 ± 37.2	57.2 ± 9.3
Green leafy vegetables (g)	39.2 ± 6.1	47.6 ± 4.9	16.2 ± 4.7
Oils and fats (g)	8.5 ± 1.7	8.6 ± 2.3	6.5 ± 1.0
Sugar (g)	10 ± 0.0	10.6 ± 1.2	6.5 ± 1.7
Nuts (g)	51.9 ± 2.1	53.0 ± 5.6	-
Fruits (g)	8.7 ± 0.5	9 ± 0.0	5.3 ± 0.5

## Supplementary Materials

The following supporting information can be downloaded at: https://www.mdpi.com/article/10.3390/nu14010225/s1, Figure S1: Funnel plot for the studies conducted to test the effect of millet vs. regular rice based meals on height; Figure S2: Funnel plot for the studies conducted to test the effect of millet vs. regular rice based meals on weight; Figure S3: Funnel plot for the studies conducted to test the effect of millet vs. regular rice based meals on MUAC; Figure S4: Funnel plot for the studies conducted to test the effect of millet vs. regular rice based meals on chest circumference; Figure S5: Funnel plot for the studies conducted to test the effect of enhanced finger millet vs. enhance rice based meal on height; Figure S6: Funnel plot for the studies conducted to test the effect of enhanced finger millet vs. enhanced rice based meals on weight; Figure S7: Funnel plot for the studies conducted to test the effect of enhanced finger millet vs. enhanced rice based meals on MUAC; Figure S8: Funnel plot for the studies conducted to test the effect of enhanced finger millet vs. enhanced rice based meals on chest circumference; Figure S9: Risk of bias assessment.
