# Supplementary material for: Can Feeding a Millet-Based Diet Improve the Growth of Children?—A Systematic Review and Meta-Analysis
Source: Nutrients. 2022 Jan 5;14(1):225. doi: 10.3390/nu14010225 (PMC8747143; doi:10.3390/nu14010225)
Supplement: Supplementary file 1 [file nutrients-14-00225-s001.zip › nutrients-1420754-supplementary.pdf]

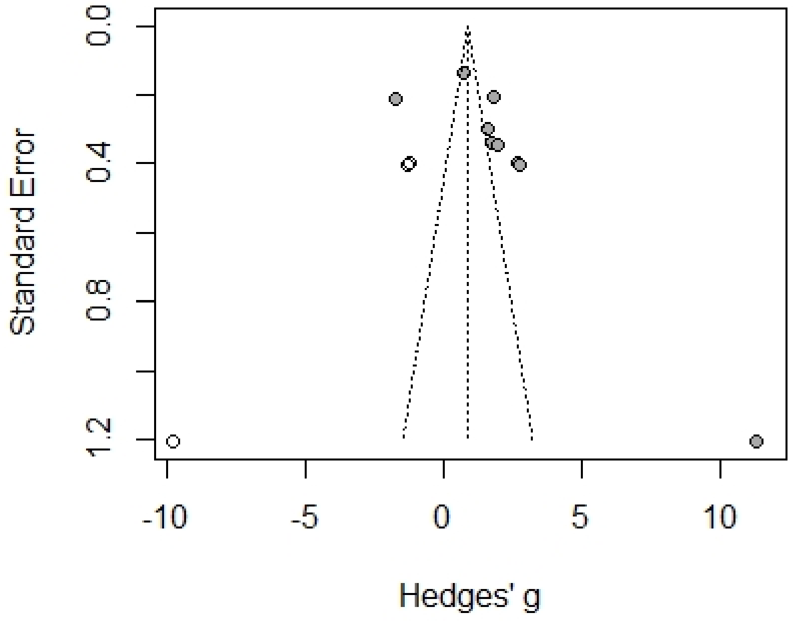

Figure S1. Funnel plot for the studies conducted to test the effect of millet vs regular rice based meals on height

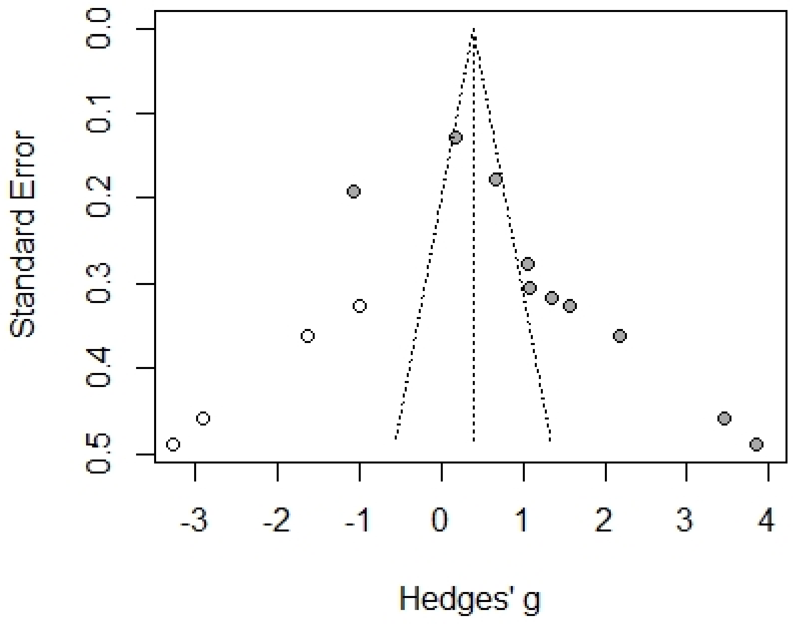

Figure S2. Funnel plot for the studies conducted to test the effect of millet vs regular rice based meals on weight

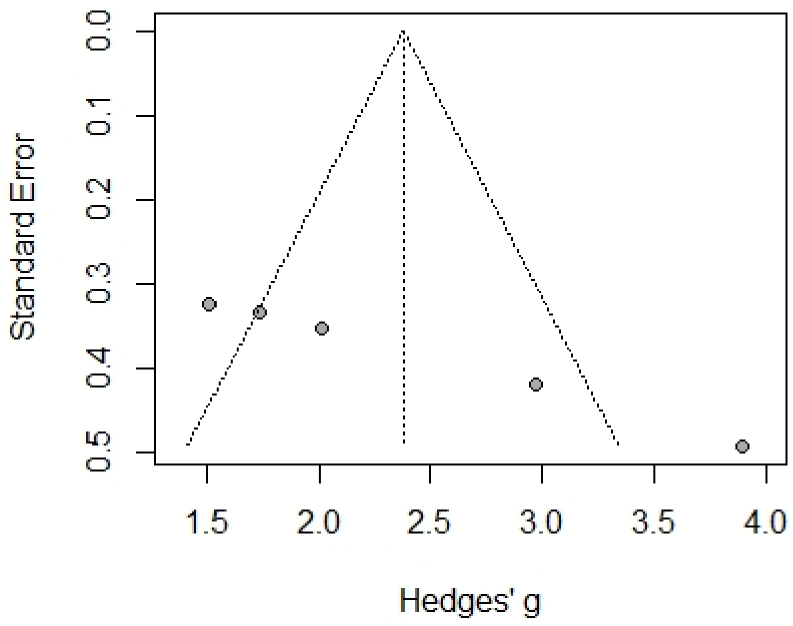

Figure S3. Funnel plot for the studies conducted to test the effect of millet vs regular rice based meals on MUAC

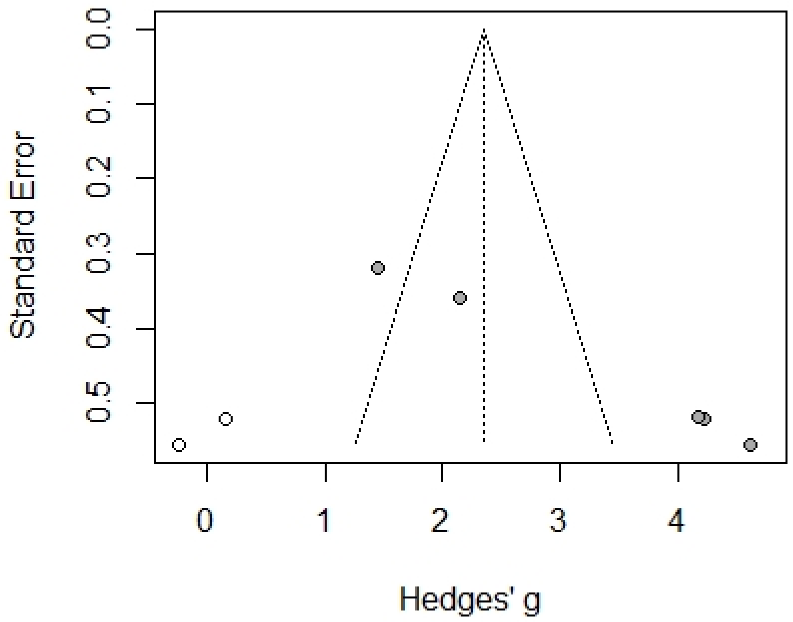

Figure S4. Funnel plot for the studies conducted to test the effect of millet vs regular rice based meals on chest circumference

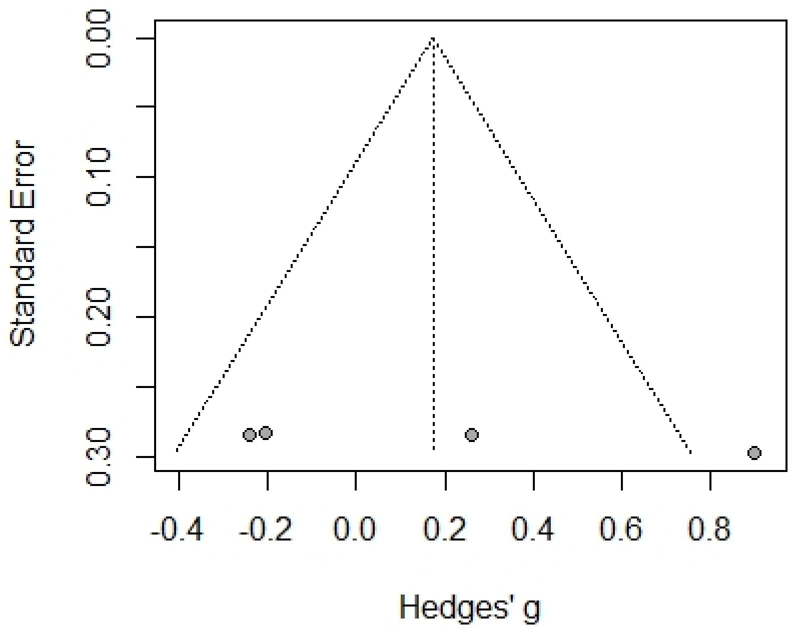

Figure S5. Funnel plot for the studies conducted to test the effect of enhanced finger millet vs enhance rice based meals on height

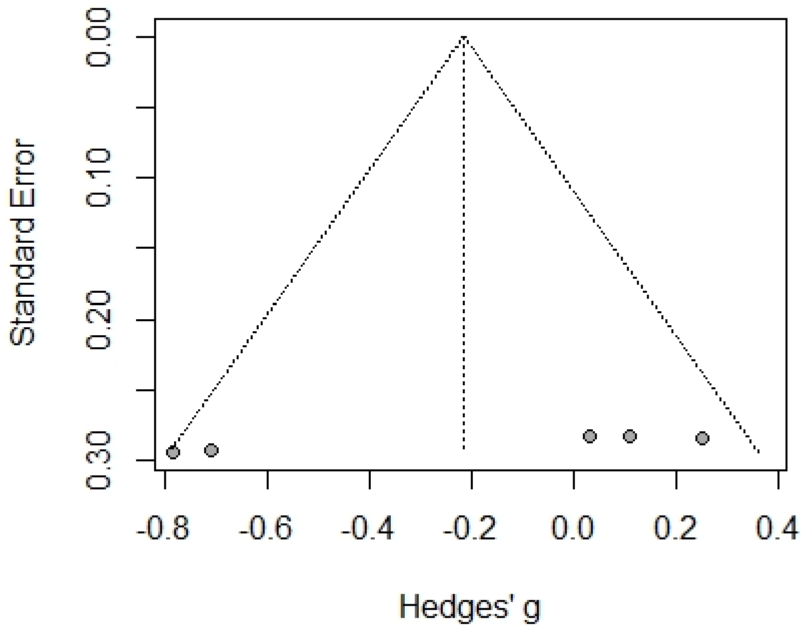

Figure S6. Funnel plot for the studies conducted to test the effect of enhanced finger millet vs enhanced rice based meals on weight

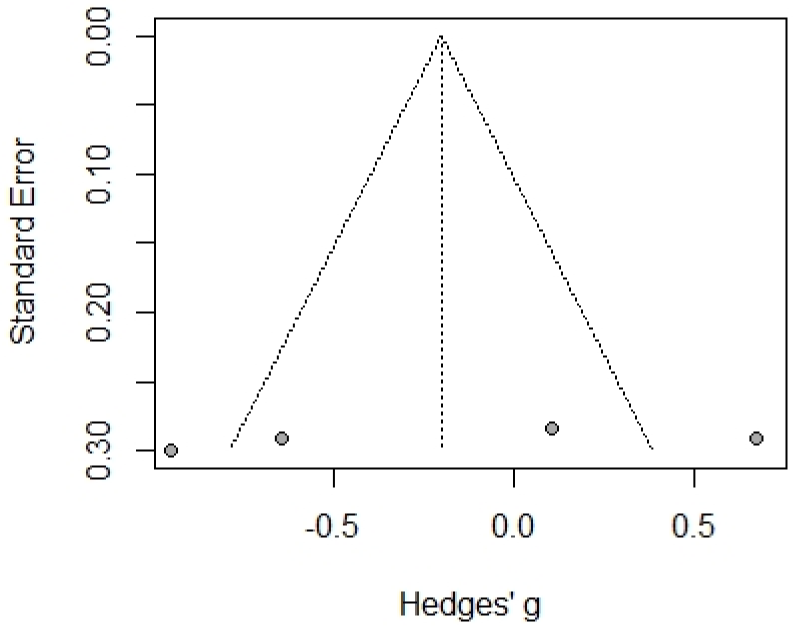

Figure S7. Funnel plot for the studies conducted to test the effect of enhanced finger millet vs enhanced rice based meals on MUAC

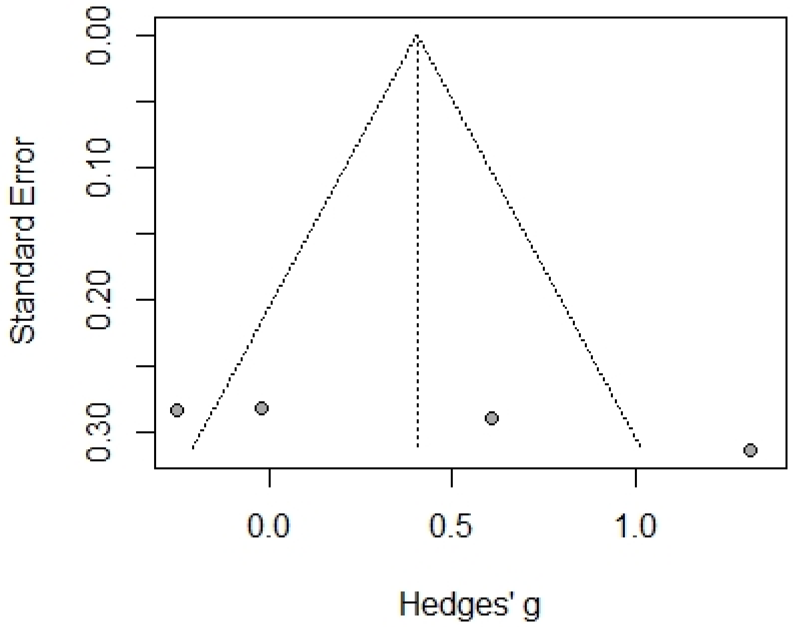

Figure S8. Funnel plot for the studies conducted to test the effect of enhanced finger millet vs modified rice based meals on chest circumference

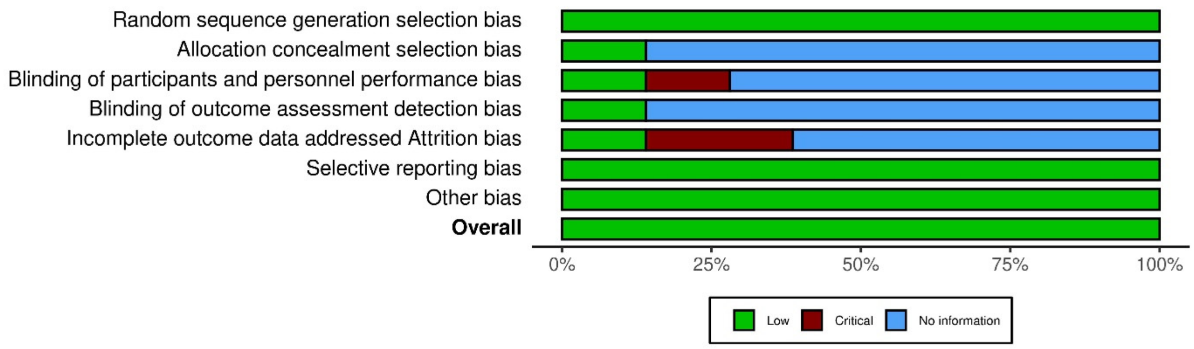

Figure S9. Risk of bias assessment
